# Males and females respond differently to treatment during isolated kidney perfusion: combined effects of glucocorticoid and estradiol

**DOI:** 10.3389/frtra.2025.1595766

**Published:** 2025-05-30

**Authors:** Marina Vidal-dos-Santos, Roberto Armstrong-Jr, Mayara Munhoz de Assis Ramos, Lucas Ferreira da Anunciação, Fernanda Yamamoto Ricardo-da-Silva, Cristiano de Jesus Correia, Petra J. Ottens, Luiz Felipe Pinho Moreira, Henri G. D. Leuvenink, Ana Cristina Breithaupt-Faloppa

**Affiliations:** ^1^ Laboratorio de Cirurgia Cardiovascular e Fisiopatologia da Circulação (LIM-11), Instituto do Coração (InCor), Faculdade de Medicina da Universidade de São Paulo, São Paulo, Brazil; ^2^ Department of Surgery, University Medical Centre Groningen, University of Groningen, Groningen, Netherlands

**Keywords:** brain death, sex differences, machine perfusion, kidney, rat

## Abstract

**Background:**

Kidney perfusion is a tool that allows organs to be assessed before transplantation. After brain death (BD), hormonal dysfunction can compromise graft quality. Hormonal treatment of donors has shown positive outcomes, and treatment during ex vivo perfusion may be advantageous. The combination of 17β-estradiol (E2) and methylprednisolone (MP) has been shown to modulate inflammation in donors. Therefore, this study aims to evaluate treatment with E2 and MP during isolated perfusion of kidneys in brain-dead male and female rats.

**Methods:**

Female and male Wistar rats were submitted to BD and maintained for 4 h. In the same animal, the right kidney [RK—no isolated perfusion of kidney (IPK)] was removed and stored, while the left kidney (LK—with IPK) had the ureter and the renal artery cannulated and flushed with 5 ml of cold saline. The LK was then taken directly to the IPK system for 90 min. Experimental groups were performed in both male and female: IPK (without treatment) and IPK + Treat (MP and E2 added to the perfusate). Perfusion was performed with a constant pressure of 100 mmHg, using William's Medium E supplemented with HEPES, creatinine, and albumin as perfusate. Perfusate and urine were collected, and flow measurements were recorded. After IPK, the LK was stored.

**Results:**

IL-6 was reduced in all perfused groups, regardless of treatment. In female IPK + Treat, there was a reduction in perfusion flow, followed by reduced creatinine clearance and Na^+^ excretion. No difference was observed in males in regards to treatment.

**Conclusion:**

The combined treatment of E2 and MP during isolated kidney perfusion compromised kidney function in females. In males, no detrimental effects were observed. These results show a sex-dependent action of the proposed treatment.

## Background

In kidney transplantation, the majority of donations are derived from brain-dead patients (1). Brain death (BD) results from cranial trauma or hemorrhagic stroke that leads to an excessive increase in intracranial pressure, culminating in herniation of the brainstem and subsequent ischemia of the brain. This process triggers a hemodynamic imbalance via the release of catecholamines, resulting in systemic inflammation and hormonal dysfunction (2).

Our group has previously shown sex differences in an experimental model of BD, with females presenting a more exacerbated inflammatory response to BD induction (3). Additionally, a study by Armstrong-Jr et al. (4) has shown that sex differences persist during ex vivo perfusion of kidneys. The use of a perfusion system is a tool to assess and treat organs in a controlled and isolated environment before transplantation, and several therapeutic options have been explored to improve graft quality (5–7). Compared with methylprednisolone (MP) administration alone, the combination of 17β-estradiol (E2) and MP improved donor hemodynamics and modulated leukocyte infiltration into the lungs (8) and kidneys (unpublished data) in an experimental model of BD in females. These results point to a positive outcome of MP and E2 administration in the donor and in an isolated perfusion system in different organs. Therefore, we aimed to investigate how the association of MP and E2 could affect kidneys from male and female rats after a period of BD using a model of isolated perfusion of kidneys (IPK).

## Methods

### Animals

Female and male Wistar rats (8–12 weeks old) from Envigo (the Netherlands) were maintained at 23 ± 2°C, with a 12 h light and dark cycle and food and water *ad libitum*. The animals were cared for in accordance with the Principles of Laboratory Animal Care (NIH Publication No. 86-23, revised 1985) and the Dutch Law on Experimental Animals Care. This study was approved by the Institutional Animal Care and Use Committee of the University of Groningen (IvD number: 171245-01-004).

The animals were allocated into groups: male and female naive, control animals that did not undergo any surgical procedure (*n* = 4); male and female BD, animals that underwent BD induction without IPK (*n* = 8); male and female IPK, animals that underwent BD, followed by IPK without treatment (*n* = 8); and male and female IPK + Treat, animals that underwent BD, followed by IPK with the combined treatment of 17β-estradiol and methylprednisolone (*n* = 8).

### Estrous cycle identification

The female animals were selected by estral cycle phase, identifying the higher estradiol period (estrous and proestrous). Vaginal lavage was performed with a Pasteur pipette filled with 10 μl of saline solution (0.9% NaCl). The cellular profile was identified by optical microscopy observation.

### Brain death induction

All animals were placed in a closed chamber, anesthetized with 5% isoflurane, and maintained with 2% isoflurane during the surgical procedure. The temperature was maintained at 37°C via a heating mat. The jugular vein was cannulated for fluid administration and influx of vasoactive drugs for hemodynamic stabilization; the carotid artery was used for blood pressure measurements. The animals were connected to a small animal ventilator after tracheostomy (Harvard Apparatus, model 683, USA) and kept at a frequency of 70 breaths/min and a tidal volume of 10 ml/kg. For BD induction, a Fogarty 4F catheter was inserted into the intracranial space and slowly inflated for 30 min. BD was confirmed by bilateral mydriasis and apnea. After BD confirmation, anesthesia was stopped, and fluid administration was initiated (2 ml/h) until the end of the experiment. Blood samples were collected at the beginning and the end of the 4 h period. After 4 h, the animals were exsanguinated, and a whole-body flush (maximum pressure of 30 mmHg) was performed with 40 ml of cold saline. Blood, urine, and organs were collected. The left kidney (LK) was prepared for perfusion, while the right kidney was immediately snap-frozen. For the LK, a nephrectomy was performed. The ureter and the renal artery were cannulated, and the kidney was then flushed with 5 ml of cold saline solution and directly transferred to the isolated kidney perfusion (IPK) system.

### Normothermic isolated perfused kidney setup

For the IPK, pressure-controlled normothermic machine perfusion was performed via a roller pump (Ismatec ISM404, Switzerland). The pressure was constantly set at 100 mmHg, and a pressure sensor (Edwards Lifesciences, USA) was placed close to the renal artery. For the perfusion solution, 100 ml of William's medium E supplemented with 30 mmol/L HEPES, 7 mmol/L creatinine, and 50 g/L albumin (Sigma-Aldrich, USA) was used. Carbogen (95% O_2_, 5% CO_2_) was used to oxygenate the perfusate at a flow rate of 0.5 L/min. Perfusate gas analyses were performed at the initial and final points of IPK, and the values were maintained at approximately 525 mmHg. A water bath and heat exchanger were used to heat the solution. In the first 30 min of perfusion, temperature was slowly increased (1°C/2 min) during the rewarming phase. After that, the temperature was kept at 37°C. Flow measurements were recorded during perfusion.

Perfusate and ultrafiltrate (urine) samples were collected at T15, T30, T60, and T90 (Tmin). After 90 min, the LK was stored for further analysis.

### Treatment

For treatment, 40 mg of methylprednisolone and 5 μg/ml of 17β-estradiol were added to the IPK perfusion solutions.

### Biochemical analysis

lactate dehydrogenase (LDH) was measured in the IPK perfusate. Creatinine and Na^+^ levels were determined in the IPK perfusate and ultrafiltrate. Measurements were performed in accordance with the Clinical Laboratory, University Medical Center Groningen, following standard biochemical methods.

### Creatinine clearance and Na excretion (FENa^+^)

The creatinine and Na^+^ concentrations in the ultrafiltrate and perfusate obtained via biochemical analysis were used to estimate creatinine clearance and Na^+^ excretion via the following calculations:

$$\begin{array}{c} \mathrm{Creatinineclearance}\;=\;(\mathrm{ultrafililtrate}\;\mathrm{creatinine}\;\mathrm{concentration}\;[\mu \mathrm{mol}/L]\;\;\times \\ \;\mathrm{ultrafiltrate}\;\mathrm{production}\\ \mathrm{flow}[\mathrm{ml}/\min]/\mathrm{perfusion}\;\mathrm{solution}\;\mathrm{creatinine}\;\mathrm{concentration}\;[\mu \mathrm{mol}/L])\\ /\mathrm{kidney}\;\mathrm{weight}\;[g]). \end{array}$$

$$\begin{array}{rl} \mathrm{FEN}a^{+}(\text{\%})\;= & \;(\mathrm{ultrafiltrate}\;Na^{\;+\;}[\mathrm{mmol}/L]\;\;\times \;\mathrm{creatinine}\;\mathrm{perfusate}\;\\ & [\mathrm{mmol}/L]/\mathrm{perfusate}\;Na^{\;+\;}[\mathrm{mmol}/L]\;\;\times \\ & \mathrm{ultrafiltrate}\;\mathrm{creatinine}\;[\mathrm{mmol}/L])\;\;\times \;100 \end{array}$$

### IL-6 quantification

IL-6 was measured in kidney homogenate and IPK perfusate. Quantifications were performed via Duo Set commercial ELISA kits (R&D Systems, USA) in accordance with the manufacturer's specifications.

### Gene expression

Total RNA was extracted from kidney tissue via TRIzol reagent (Invitrogen, USA). The yield of extracted RNA was analyzed with a NanoDrop 1000 spectrophotometer (NanoDrop Technologies, USA), and the quality was assessed via RNA electrophoresis. The extracted RNA was reverse transcribed with random primers at 37°C for 50 min. Quantitative real-time polymerase chain reaction (qRT-PCR) was conducted with specific primers (Table 1). SYBR Green (Applied Biosystems, the Netherlands) and a QuantStudio 7 Flex qPCR machine (Applied Biosystems, the Netherlands), which was configured with 1 cycle of 10 min at 95°C and 40 consecutive cycles of 15 s at 95°C and 1 min at 60°C. CT values were corrected for β-actin levels.

**Table 1:** RT-PCR primers.

Gene	Forward sequence	Reverse sequence
β-Actin	GGAAATCGTGCGTGACATTAAA	GCGGCAGTGGCCATCTC
IL-6	CCAACTTCCAATGCTCTCCTAATG	TTCAAGTGCTTTCAAGAGTTGGAT
KIM-1	AGAGAGAGCAGGACACAGGCTTT	ACCCGTGGTAGTCCCAAACA

RT-PCR, real-time polymerase chain reaction; β-actin, beta-actin; IL-6, interleukin 6; KIM-1, kidney injury marker 1.

### Immunohistochemistry

For the immunohistochemical analyses, the right and left kidneys were placed in formalin and subsequently paraffin-embedded. Slices (3 mm) were prepared and placed on glass slides. Deparaffinization was performed via xylene and ethanol. Antigen retrieval was performed via the use of an ethylenediaminetetraacetic acid (EDTA) (pH 8) solution. The slides were incubated in this solution for 3 h at 60°C. Later, endogenous peroxidase was blocked with H2O2 (2%) for 15 min. Non-specific blockage was performed with TBST-BSA (2%) solution for 1 h at 37°C. Antibodies against myeloperoxidase (MPO), inducible nitric oxide synthase (iNOS), kidney injury molecule-1 (KIM-1), endothelial nitric oxide synthase (eNOS), and endothelin-1 (ET-1) (Table 2) were incubated with the samples overnight at 4°C. The sections were then incubated with secondary HRP-conjugated antibodies (1:200, BA1054; Boster Biological Technology, USA) for 2 h at 37°C. A peroxidase substrate was subsequently used. Hematoxylin was used for counterstaining. For the slide analyses and image acquisition, NIS-Elements BD (Nikon, Japan) software was used.

**Table 2:** Primary antibodies.

Antibody	Dilution and code
MPO	1:50, PA1054; Boster Biological Technology, USA
iNOS	1:100, AB3523; Abcam, UK
KIM-1	1:100, LS-C312791; LSBio, USA
eNOS	1:100, AO1604-2; Boster Biological Technology, USA
Endothelin-1	1:200, LS-B13907; LSBio, USA

MPO, myeloperoxidase; iNOS, inducible nitric oxide synthase; KIM-1, kidney injury marker 1; eNOS, endothelial nitric oxide synthase.

### Morphological analysis

Kidney morphology was analyzed via periodic acid–Schiff (PAS) staining of paraffin-embedded kidney sections (5 μm). The scoring system used is presented in Table 3.

**Table 3:** Morphological analysis scoring system.

Analyses	Scoring system			
	0	1	2	3
Dilated glomerulus	Normal glomerular space	Moderately enlarged glomerular space	Enlarged glomerular space	Greatly enlarged glomerular space
Necrosis of the proximal tubule	–	Partly broken brush border, shedding and blebbing, and normal nucleus	>50% broken brush border, loose cell nuclei, and pyknotic nuclei	More than half of the tubules are necrotic
Necrosis of the distal tubule	–	Partly loose cells	>50% loose cells and pyknotic nuclei	–

### Statistical analysis

The data are expressed as the means ± standard errors of the means (SEMs). The data were analyzed with GraphPad Prism Version 10, and the groups were compared via two-way analysis followed by the *post hoc* test of the two-stage linear step-up procedure of Benjamin, Krieger, and Yekutieli or three-way ANOVA.

## Results

### Expression and release of IL-6

**Figure 1:**
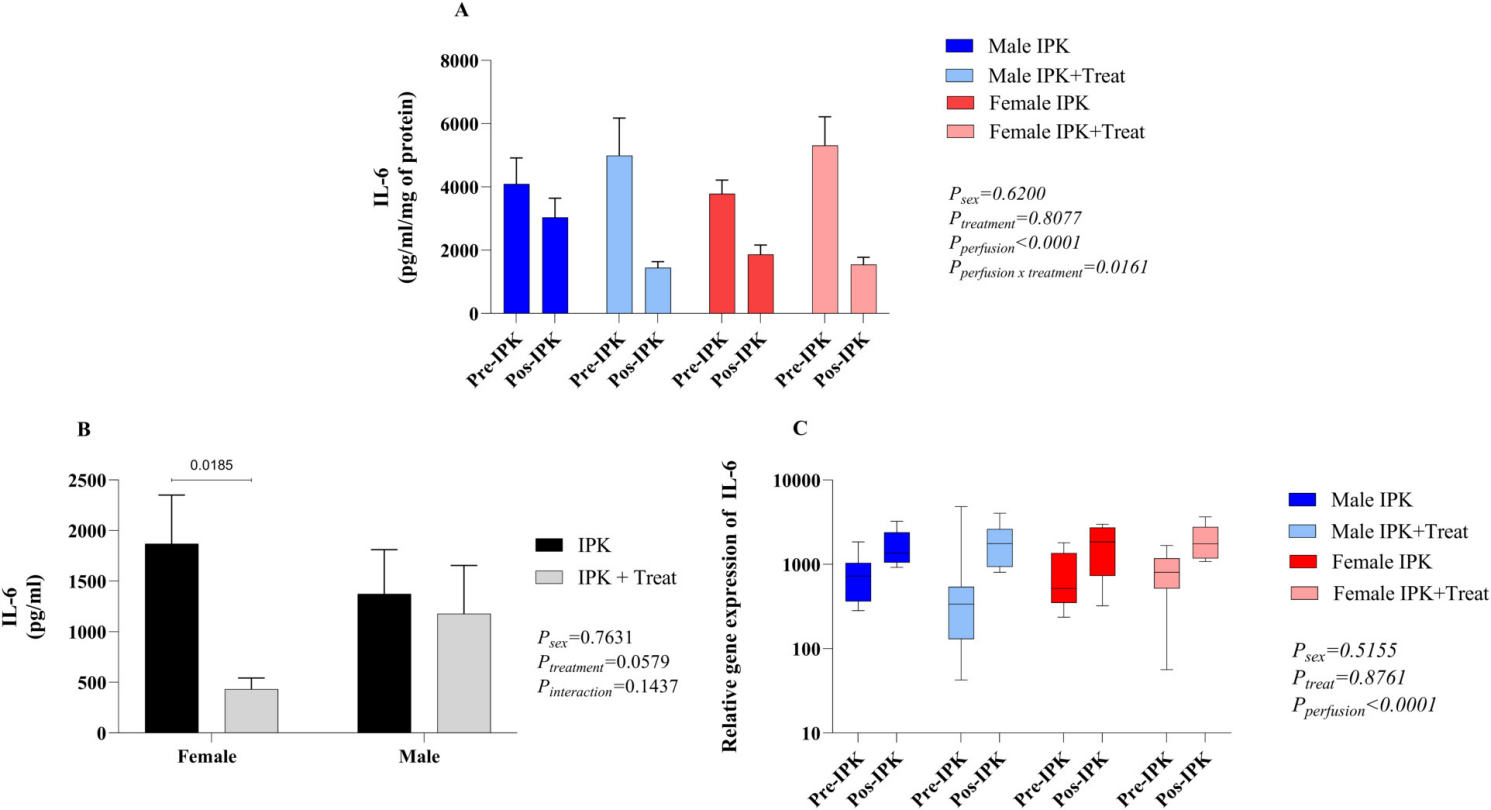
Quantification of IL-6 in kidney homogenate **(A)**, IPK perfusate **(B)**, and gene expression in kidney tissue **(C)**. IPK, kidneys that underwent isolated perfusion without treatment; IPK + Treat, kidneys that underwent isolated perfusion with treatment of 17β-estradiol and methylprednisolone. Results are presented as mean and standard error of the mean. Results from graphs A and C were analyzed using three-way ANOVA. Results from graph B were analyzed using two-way ANOVA. IL-6, interleukin 6.

IL-6 (Figure 1) was quantified in kidney homogenates (A) and IPK perfusates (B). Gene expression in renal tissue was also evaluated (C). Compared with non-perfused kidneys, all groups presented reduced expression of IL-6 in kidney homogenates after perfusion. For males, the treated group presented even lower concentrations than those in the non-treated group. In females, no difference was observed between the IPK and IPK + Treat groups (A). According to the results of the perfusate analyses, female IPK + Treat mice presented lower release of IL-6 than IPK-treated mice did. No difference was observed in males (B). Finally, gene expression was increased in all groups after IPK, regardless of sex and treatment (C).

### Leukocyte infiltrate

**Figure 2:**
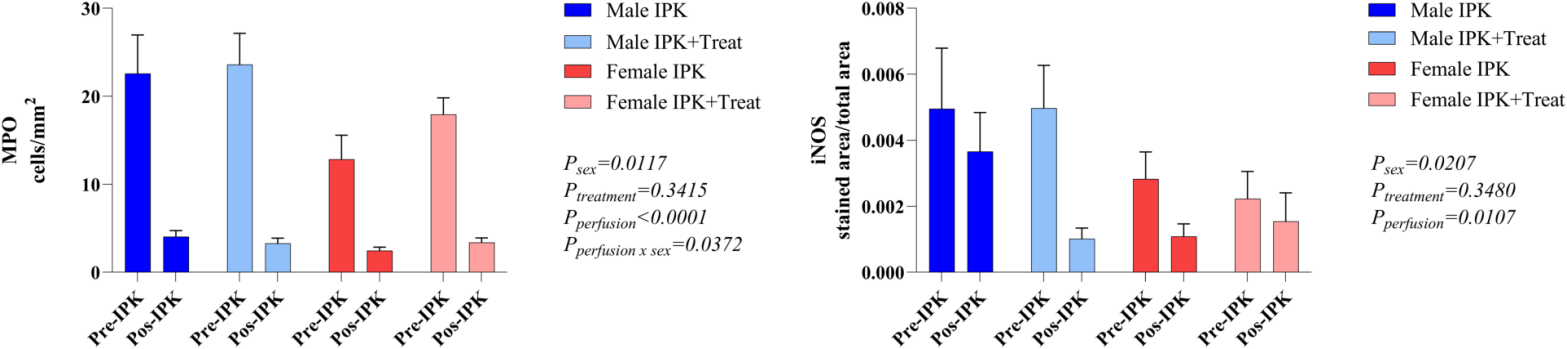
Protein expression of MPO and iNOS in kidney tissue. IPK, kidneys that underwent isolated perfusion without treatment; IPK + Treat, kidneys that underwent isolated perfusion with treatment of 17β-estradiol and methylprednisolone. Results are presented as mean and standard error of the mean. Results analyzed using three-way ANOVA. MPO, myeloperoxidase. iNOS, inducible nitric oxide synthase.

The number of infiltrating neutrophils and macrophages was analyzed via quantification of MPO-marked cells and iNOS protein expression (Figure 2). The pre-IPK kidneys of males presented higher MPO levels than did those of females. After IPK, all groups presented similar numbers of infiltrated cells (A). Similarly, in the iNOS analyses, protein expression was increased in males and was reduced after perfusion in all the groups.

### Kidney injury marker 1 (KIM-1) expression

**Figure 3:**
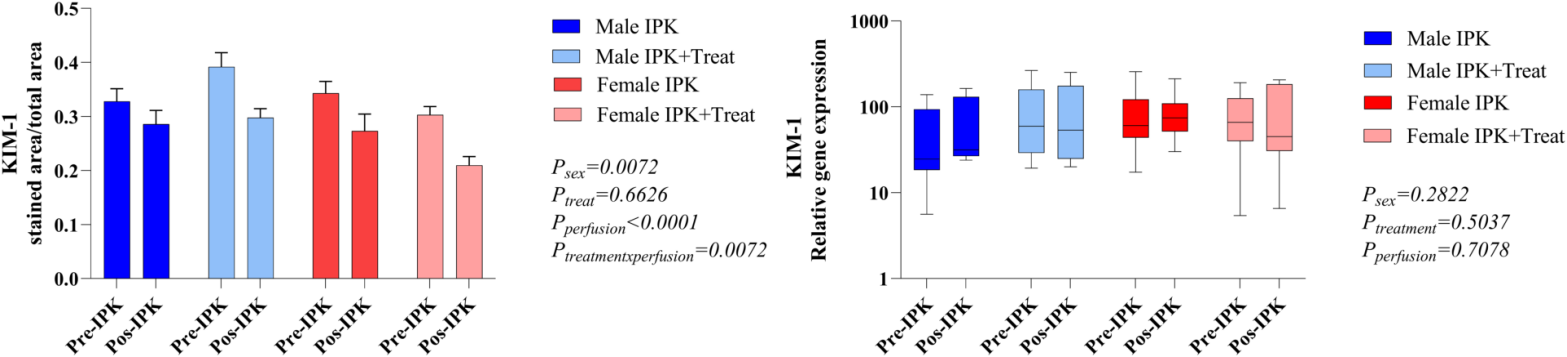
Protein and gene expression of KIM-1 in kidney tissue. IPK, kidneys that underwent isolated perfusion without treatment; IPK + Treat, kidneys that underwent isolated perfusion with treatment of 17β-estradiol and methylprednisolone. Results are presented as mean and standard error of the mean. Results analyzed using three-way ANOVA. KIM-1, kidney injury marker 1.

There was a reduction in the protein expression of KIM-1 with perfusion. With respect to gene expression, no difference was observed among the groups (Figure 3).

### Flow measurement

**Figure 4:**
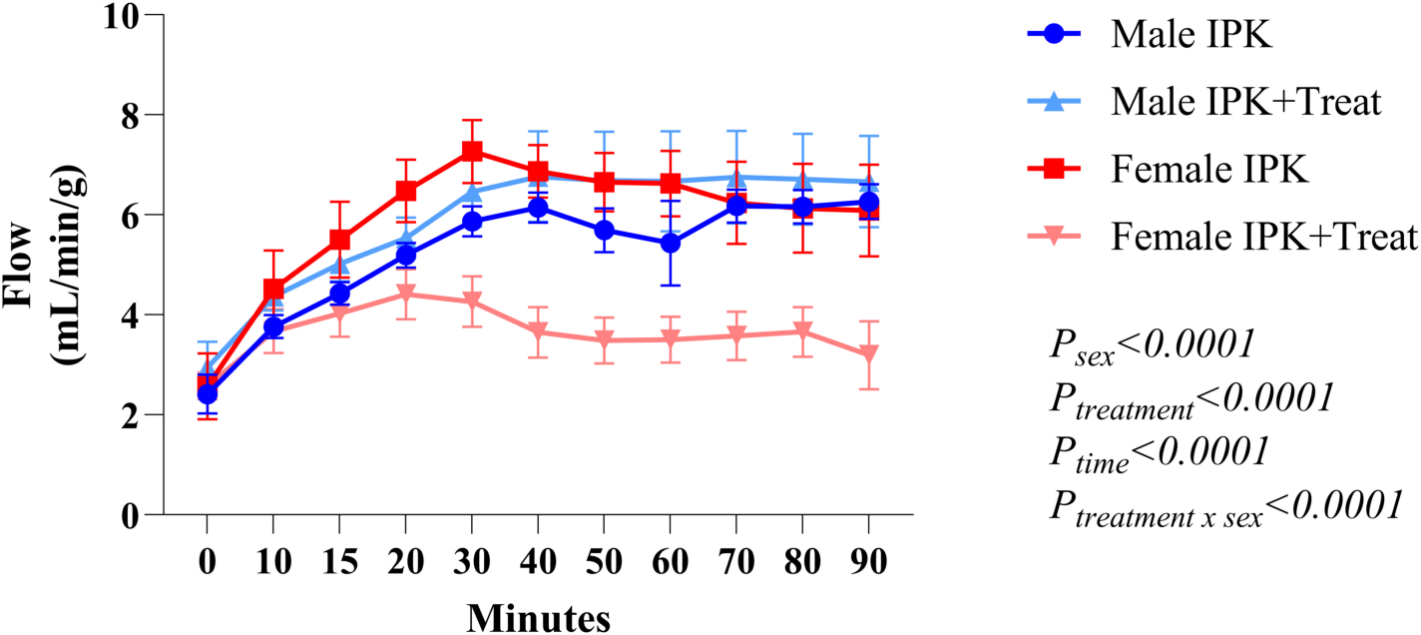
Flow measurement during perfusion. IPK, kidneys that underwent isolated perfusion without treatment; IPK + Treat, kidneys that underwent isolated perfusion with treatment of 17β-estradiol and methylprednisolone. Results are presented as mean and standard error of the mean. Results analyzed using three-way ANOVA.

Perfusion flow was constantly measured during IPK. Females presented with slightly increased flow at the beginning of perfusion. Compared with the control group, the IPK + Treat group presented significantly reduced flow. The male groups presented similar values throughout perfusion (Figure 4).

### Modulation of vascular tone

**Figure 5:**
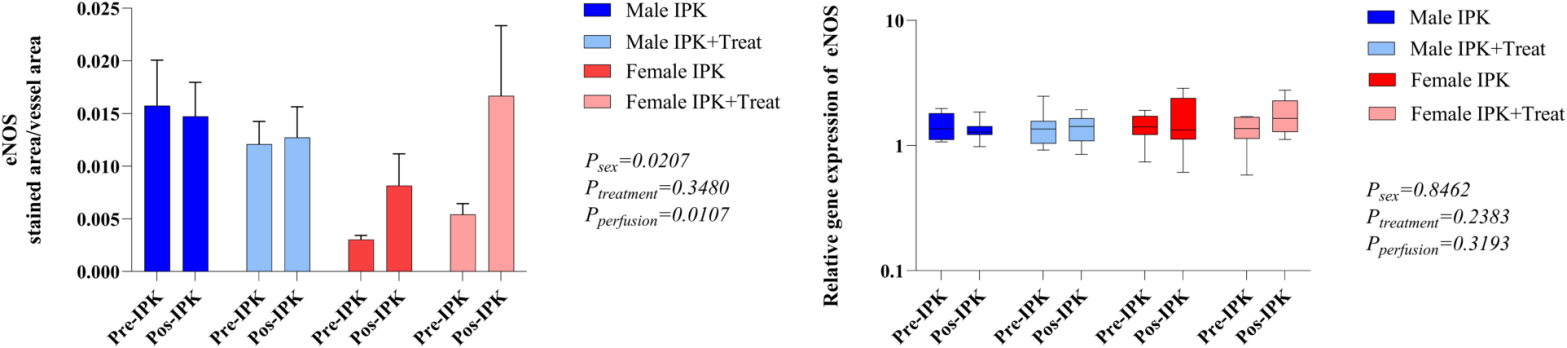
Protein expression of eNOS and endothelin-1 in kidney tissue. IPK, kidneys that underwent isolated perfusion without treatment; IPK + Treat, kidneys that underwent isolated perfusion with treatment of 17β-estradiol and methylprednisolone. Results are presented as mean and standard error of the mean. Results analyzed using three-way ANOVA. eNOS, endothelial nitric oxide synthase.

The protein expression of eNOS and endothelin-1 (ET-1) was analyzed in kidney tissue (Figure 5). In females, eNOS expression was reduced in pre-IPK kidneys, and eNOS expression was increased in the IPK + Treat group (A). No difference was observed in the male groups. With respect to ET-1 (B), females also presented reduced expression in pre-IPK kidneys compared with males, and no difference was observed with perfusion or treatment.

### Kidney function

**Figure 6:**
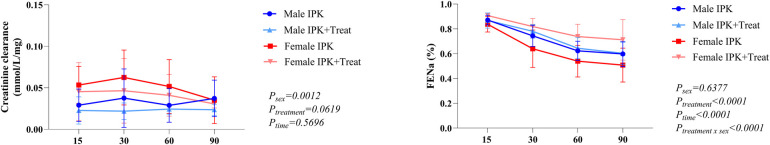
Creatinine clearance and fractional excretion of sodium. IPK, kidneys that underwent isolated perfusion without treatment; IPK + Treat, kidneys that underwent isolated perfusion with treatment of 17β-estradiol and methylprednisolone. Results are presented as mean and standard error of the mean. Results analyzed using three-way ANOVA. FENa, fractional excretion of sodium.

Creatinine clearance was lower in males than in females, and female IPK + Treat presents lower values than female IPK does. No difference was observed over time (A). Fractional excretion of sodium (FENa) in the perfusate revealed greater reabsorption of Na^+^ in the female IPK + Treat group than in the female IPK group. Both male groups presented similar values at all 90 min (B). Over time, all groups presented a reduction in FENa^+^ (Figure 6).

### Lactate dehydrogenase (LDH) and lactate

**Figure 7:**
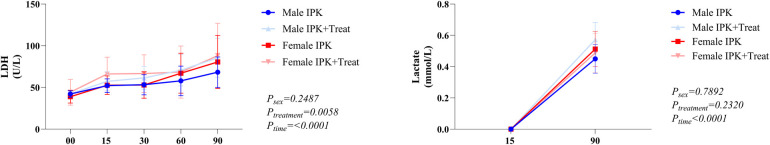
Lactate dehydrogenase and lactate quantification. IPK, kidneys that underwent isolated perfusion without treatment; IPK + Treat, kidneys that underwent isolated perfusion with treatment of 17β-estradiol and methylprednisolone. Results are presented as mean and standard error of the mean. Results analyzed using three-way ANOVA. LDH, lactate dehydrogenase.

LDH was measured in the perfusate at several time points (A). Lactate was quantified at the beginning and end of the perfusion (B). There is an increase in LDH in both male and female IPK + Treat groups compared with IPK. All groups presented an increase in LDH throughout perfusion. Lastly, all groups presented increased lactate from T15 to T90 (Figure 7).

### Morphological analysis of renal tissue

**Figure 8:**
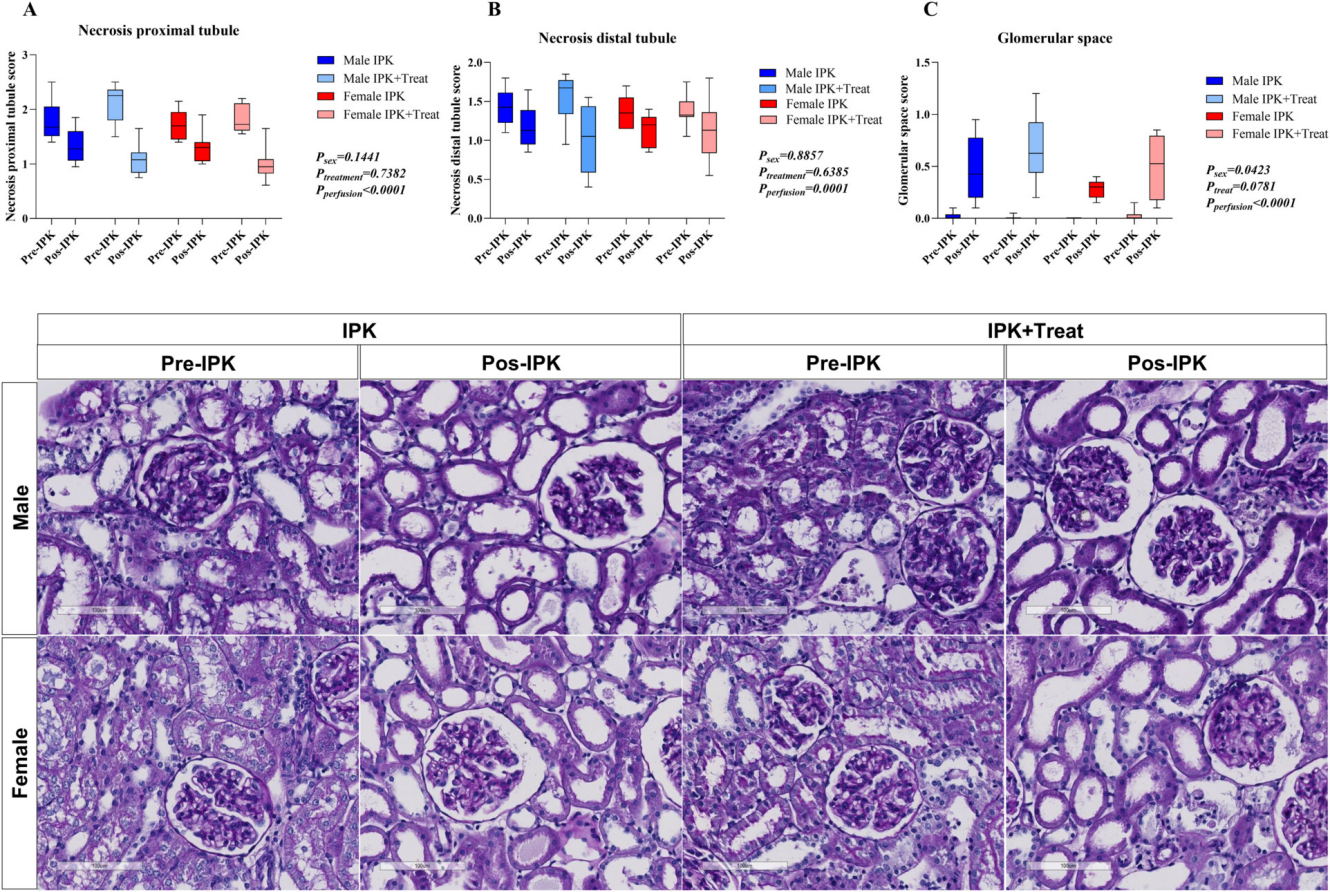
Morphological analysis of kidney tissue. Necrosis of the proximal tubule **(A)**, necrosis of the distal tubule **(B)**, and glomerular space **(C)**. IPK, kidneys that underwent isolated perfusion without treatment; IPK + Treat, kidneys that underwent isolated perfusion with treatment of 17β-estradiol and methylprednisolone. Results are presented as mean and standard error of the mean. Results were analyzed using three-way ANOVA. Representative images are presented (20×).

Necrosis of the proximal and distal tubules and glomerular space was quantified via a scoring system (Figure 8). Both proximal and distal necrosis were reduced after perfusion in all groups (A and B). Compared with non-perfused kidneys, all perfused kidneys presented increased glomerular space (C).

## Discussion

We investigated the effects of E2 and MP on kidney injury during normothermic isolated perfusion to improve graft quality. Previously, Armstrong-Jr et al. (4) highlighted sex differences during perfusion using the same experimental BD model. Their study revealed increased cellular infiltration in males, especially of macrophages, and reduced creatinine clearance compared with females. In our work, we observed a reduction in inflammation in all perfused groups, regardless of treatment and sex, with decreased protein expression of IL-6 in kidney tissues and a reduced presence of leukocytes in the parenchyma. In terms of treatment, female IPK + Treat presented reduced flow compared with female IPK, accompanied by decreased creatinine clearance and increased fractional excretion of sodium, whereas males presented similar renal function results in the treated and non-treated groups.

The first clinical ex vivo kidney perfusion was performed in 2011 to serve as a platform to preserve, assess, and repair kidneys before transplantation (9). Several protocols have been tested to provide a near-physiological environment (10), and studies have linked normothermic kidney perfusion (NKP) to inflammation (11). To date, there is no consensus on the appropriate perfusion conditions and ideal biomarkers for evaluating graft quality. Perfusion solutions usually include red blood cells (RBCs) or even whole blood as oxygen carriers (12), and such compositions can lead to complement activation, even with the use of leukocyte filters or plasma-free perfusate (13). De Beule et al. (14) compared RBCs and whole blood-based solutions during the NKP of ischemic and nonischemic kidneys. Their results point to increased renal damage, with reduced oxygen consumption and increased levels of injury markers in kidneys that underwent warm and cold ischemia periods, with no differences related to perfusate type. These results suggest that increased inflammation during NKP may also be related to reperfusion injury triggered by blood-derived products. In our study, we observed a reduced IL-6 concentration in kidney tissue after perfusion and a reduced presence of infiltrating neutrophils. These findings could be related to the use of RBC-free perfusate, which mitigates the inflammatory response triggered by blood components. No evidence is available on ideal oxygenation during NKP, but most experimental protocols use supraphysiological concentrations, and even though no oxygen carrier was used, our perfusion solution was able to provide pO_2_ values above 500 mmHg.

Additionally, perfusion flow was evaluated throughout the 90 min of perfusion. In treated females, reduced flow was reflected in lower creatinine clearance rates and increased FENa^+^. FENa^+^ is calculated to assess sodium handling by the kidney and is used to evaluate acute kidney injury. Usually, values <1% may suggest prerenal diseases; however, greater reabsorption of Na^+^ represents an appropriate response to reduced kidney perfusion (15). In a canine model, Dvorak et al. (16) evaluated the administration of several doses of MP during isolated perfusion of kidneys from female dogs and reported that high-dose MP (0.32 mg/ml) led to increased perfusion pressure and reduced perfusion flow. In our study, a similar dose was used (0.40 mg/ml), which could explain the reduction in flow observed in the female IPK + Treat group. In women, pharmacokinetic studies focused on premenopausal kidney transplant recipients have shown that the MP clearance rate is lower in women than in men (17). The same phenomenon is observed in the physiological clearance of MPs (18). However, in the clinic, a standardized dose protocol is used, with no consideration of the age or sex of the patient (19), highlighting the importance of studies focusing on sex differences.

Additionally, activation of the mineralocorticoid receptor (MR) in the kidney by the hormone aldosterone promotes Na^+^ retention and hypertension. Although MRs present high affinity for aldosterone, other glucocorticoids, such as cortisol, can interact with and activate such receptors (20). Exogenous glucocorticoids were initially considered free of cross-activation of MR; however, Rebuffat et al. (21) reported that dexamethasone was able to occupy MR and promote its translocation to the nucleus. Several studies have shown that female mice and humans present increased expression of endothelial MRs and are at increased risk of vascular damage mediated by MR activation (22–24). In our model, MP administration in females may have activated MR in the renal endothelium, leading to vascular damage. Therefore, we also investigated the modulation of vascular tone by quantifying the protein expression of ET-1 and eNOS in kidney tissue and observed reduced expression of ET-1 in females compared with males. In the literature, studies have shown sex differences in ET-1 expression, with lower levels in females, under both physiological and pathological conditions, and such findings have been associated with higher E2 concentrations (25–27). E2 is also known to stimulate eNOS activation/expression (28, 29), and our group has previously shown that treatment of donors with E2 after BD can increase eNOS (30, 31). Although eNOS is constitutively expressed, several chemical and physical stimuli affect eNOS expression/activity. For example, fluid shear stress generated in vessels during perfusion can activate eNOS mRNA and protein expression (32). Indeed, our results revealed that females presented increased protein expression of eNOS in both perfused groups, especially in the IPK + Treat group.

This study has some limitations. Our protocol uses a small animal model, and correlation to the clinic is limited. Further studies should be conducted in larger animals or discarded human kidneys for a more translational application. Moreover, a transplantation model would also be of interest, aiming to evaluate the effect of the proposed treatment on the recipient.

In conclusion, the data presented in this study show that perfusion alone was able to ameliorate inflammation by reducing the number of infiltrated leukocytes and the expression of IL-6, as well as ameliorating kidney injury by reducing the protein expression of KIM-1 and necrosis of the distal and proximal tubules. Specifically, in males, the use of the proposed treatment had no detrimental effects during perfusion, with the analyzed parameters remaining similar. In females, treatment with E2 and MP had deleterious effects on isolated kidneys, especially by reducing perfusion flow and consequently compromising kidney function. In a parallel study (data not published) using an ex vivo lung perfusion model, the combination of E2 and MP improved lung function in males and modulated inflammation in females, indicating that treatment effectiveness might be organ- and sex-dependent. Subsequent studies should aim to evaluate both male and female subjects, as sex differences may modulate preservation techniques and treatments in positive or negative ways. Thus, personalized protocols could represent the future of graft management.

## Data Availability

The raw data supporting the conclusions of this article will be made available by the authors, without undue reservation.

## References

[1] Eurotransplant Annual Report. (2023). Available at: https://www.eurotransplant.org/statistics/annual-report/ (Accessed February 5, 2025).

[2] Westendorp WH Leuvenink HG Ploeg RJ. Brain death induced renal injury. Curr Opin Organ Transpl. (2011) 16:151–6. 10.1097/MOT.0b013e328344a5dc 21415817

[3] Breithaupt-Faloppa AC Ferreira SG Kudo GK Armstrong-Jr R Tavares-de-Lima W da Silva LF Sex-related differences in lung inflammation after brain death. J Surg Res. (2016) 200(2):714–21. 10.1016/j.jss.2015.09.018 26547667

[4] Armstrong-Jr R Ricardo-da-Silva FY Vidal-Dos-Santos M da Anunciação LF Ottens PJ Correia CJ Comparison of acute kidney injury following brain death between male and female rats. Clinics (Sao Paulo). (2023) 78:100222. 10.1016/j.clinsp.2023.100222 37257364 PMC10244907

[5] Maassen H Venema LH Weiss MG Huijink TM Hofker HS Keller AK H2S-enriched flush out does not increase donor organ quality in a porcine kidney perfusion model. Antioxidants (Basel). (2023) 12(3):749. 10.3390/antiox12030749 36978997 PMC10044751

[6] Ogurlu B Pamplona CC Van Tricht IM Hamelink TL Lantinga VA Leuvenink HGD Prolonged controlled oxygenated rewarming improves immediate tubular function and energetic recovery of porcine kidneys during normothermic machine perfusion. Transplantation. (2023) 107(3):639–47. 10.1097/TP.0000000000004427 36525548 PMC9946163

[7] Lohmann S Eijken M Møldrup U Møller BK Hunter J Moers C Ex vivo administration of mesenchymal stromal cells in kidney grafts against ischemia-reperfusion injury-effective delivery without kidney function improvement posttransplant. Transplantation. (2021) 105(3):517–28. 10.1097/TP.0000000000003429 32956281

[8] Vidal-Dos-Santos M Anunciação LF Armstrong-Jr R Ricardo-da-Silva FY Ramos IYT Correia CJ 17*β*-estradiol and methylprednisolone association as a therapeutic option to modulate lung inflammation in brain-dead female rats. Front Immunol. (2024) 15:1375943. 10.3389/fimmu.2024.1375943 38765005 PMC11099279

[9] Hosgood S Nicholson M. First in man renal transplantation after ex-vivo normothermic perfusion. Transplantation. (2011) 92:735–8. 10.1097/TP.0b013e31822d4e04 21841540

[10] Elliott TR Nicholson ML Hosgood SA. Normothermic kidney perfusion: an overview of protocols and strategies. Am J Transplant. (2021) 21(4):1382–90. 10.1111/ajt.16307 32897651

[11] Mellati A Lo Faro L Dumbill R Meertens P Rozenberg K Shaheed S Kidney normothermic machine perfusion can be used as a preservation technique and a model of reperfusion to deliver novel therapies and assess inflammation and immune activation. Front Immunol. (2022) 13:850271. 10.3389/fimmu.2022.850271 35720316 PMC9198253

[12] Hamelink TL Ogurlu B De Beule J Lantinga VA Pool MBF Venema LH Renal normothermic machine perfusion: the road toward clinical implementation of a promising pretransplant organ assessment tool. Transplantation. (2022) 106(2):268–79. 10.1097/TP.0000000000003817 33979315

[13] de Boer E Sokolova M Jager NM Schjalm C Weiss MG Liavåg OM Normothermic machine perfusion reconstitutes porcine kidney tissue metabolism but induces an inflammatory response, which is reduced by complement C5 inhibition. Transpl Int. (2024) 37:13348. 10.3389/ti.2024.13348 39606689 PMC11598510

[14] De Beule J Keppens D Korf H Jochmans I. Differential cytokine levels during normothermic kidney perfusion with whole blood- or red blood cell-based perfusates—results of a scoping review and experimental study. J Clin Med. (2022) 11:6618. 10.3390/jcm11226618 36431095 PMC9695901

[15] Okusa MD Chopra TA. Fractional excretion of sodium, urea, and other molecules in acute kidney injury. United States: Wolters Kluwer (2025). Available at: https://www.uptodate.com/contents/fractional-excretion-of-sodium-urea-and-other-molecules-in-acute-kidney-injury# (Accessed February 3, 2025).

[16] Dvorak K Braun W Magnusson M Stowe N Banowsky L. Effect of high doses of methylprednisolone on the isolated, perfused canine kidney. Transplantation. (1976) 21:149–57. 10.1097/00007890-197602000-00010 1251463

[17] Tornatore K Gilliland-Johnson K Farooqui M Reed K Venuto R. Pharmacokinetics and pharmacodynamic response of methylprednisolone in premenopausal renal transplant recipients. J Clin Pharmacol. (2004) 44:1003–11. 10.1177/0091270004268130 15317828

[18] Ayyar V DuBois D Nakamura T Almon R Jusko W. Modeling corticosteroid pharmacokinetics and pharmacodynamics, part II: sex differences in methylprednisolone pharmacokinetics and corticosterone suppression. J Pharmacol Exp Ther. (2019) 370:327–36. 10.1124/jpet.119.257527 31197019 PMC7184193

[19] Tornatore KM Biocevich DM Reed K Tousley K Singh JP Venuto RC. Methylprednisolone pharmacokinetics, cortisol response, and adverse effects in black and white renal transplant recipients. Transplantation. (1995) 59(5):729–36. 10.1097/00007890-199503150-00016 7886801

[20] Frey FJ Odermatt A Frey BM. Glucocorticoid-mediated mineralocorticoid receptor activation and hypertension. Curr Opin Nephrol Hypertens. (2004) 13(4):451–8. 10.1097/01.mnh.0000133976.32559.b0 15199296

[21] Rebuffat AG Tam S Nawrocki AR Baker ME Frey BM Frey FJ The 11-ketosteroid 11-ketodexamethasone is a glucocorticoid receptor agonist. Mol Cell Endocrinol. (2004) 214(1-2):27–37. 10.1016/j.mce.2003.11.027 15062542

[22] Faulkner JL Belin de Chantemèle EJ. Mineralocorticoid receptor and endothelial dysfunction in hypertension. Curr Hypertens Rep. (2019) 21(10):78. 10.1007/s11906-019-0981-4 31485760 PMC6878110

[23] Faulkner JL Kennard S Huby AC Antonova G Lu Q Jaffe IZ Progesterone predisposes females to obesity-associated leptin-mediated endothelial dysfunction via upregulating endothelial MR (mineralocorticoid receptor) expression. Hypertension. (2019) 74(3):678–86. 10.1161/HYPERTENSIONAHA.119.12802 31327274 PMC6687552

[24] Faulkner JL Harwood D Kennard S Antonova G Clere N Belin de Chantemèle EJ. Dietary sodium restriction sex-specifically impairs endothelial function via mineralocorticoid receptor-dependent reduction in NO bioavailability in balb/C mice. Am J Physiol Heart Circ Physiol. (2020) 320(1):H211–20. 10.1152/ajpheart.00413.2020 33095056 PMC7847080

[25] Miyauchi T Yanagisawa M Iida K Ajisaka R Suzuki N Fujino M Age- and sex-related variation of plasma endothelin-1 concentration in normal and hypertensive subjects. Am Heart J. (1992) 123(4 Pt 1):1092–3. 10.1016/0002-8703(92)90734-d 1549986

[26] Polderman KH Stehouwer CD van Kamp GJ Dekker GA Verheugt FW Gooren LJ. Influence of sex hormones on plasma endothelin levels. Ann Intern Med. (1993) 118(6):429–32. 10.7326/0003-4819-118-6-199303150-00006 8439117

[27] Polderman KH Stehouwer CD van Kamp GJ Schalkwijk CG Gooren LJ. Modulation of plasma endothelin levels by the menstrual cycle. Metabolism. (2000) 49(5):648–50. 10.1016/s0026-0495(00)80042-6 10831177

[28] Goetz R Thatte H Prabhakar P Cho M Michel T Golan D. Estradiol induces the calcium-dependent translocation of endothelial nitric oxide synthase. Proc Natl Acad Sci U S A. (1999) 96(6):2788–93. 10.1073/PNAS.96.6.2788 10077589 PMC15847

[29] Musicki B Liu T Strong T Lagoda G Bivalacqua T Burnett A. Post-translational regulation of endothelial nitric oxide synthase (eNOS) by estrogens in the rat vagina. J Sex Med. (2010) 7(5):1768–77. 10.1111/j.1743-6109.2010.01750.x 20233295 PMC2884058

[30] Armstrong-Jr R Ricardo-da-Silva FY Correia CJ Vidal-Dos-Santos M da Anunciação LF Coutinho E Silva RS Treatment with 17*β*-estradiol protects donor heart against brain death effects in female rat. Transpl Int. (2020) 33(10):1312–21. 10.1111/tri.13687 32621784

[31] Vieira RF Breithaupt-Faloppa AC Matsubara BC Rodrigues G Sanches MP Armstrong-Jr R 17*β*-estradiol protects against lung injuries after brain death in male rats. J Heart Lung Transplant. (2018 Nov) 37(11):1381–7. 10.1016/j.healun.2018.06.015 30139547

[32] Balligand JL Feron O Dessy C. eNOS activation by physical forces: from short-term regulation of contraction to chronic remodeling of cardiovascular tissues. Physiol Rev. (2009) 89(2):481–534. 10.1152/physrev.00042.2007 19342613

